# Biomarkers as Temporal Signals: A Decision-Linked Multi-Layer Framework for Exercise Recovery, Overload, and Adaptation

**DOI:** 10.3390/ijms27083675

**Published:** 2026-04-20

**Authors:** Dan Cristian Mănescu, Camelia Daniela Plăstoi, Ancuța Pîrvan, Cristina Daniela Pașcan, Lucian Păun, Ionuț Eduard Sersea, Bogdan Niculescu, Viorela Elena Popescu, Andreea Voinea, Andreea Popescu

**Affiliations:** 1 Department of Physical Education and Sport, Bucharest University of Economic Studies, 010374 Bucharest, Romania; dan.manescu@defs.ase.ro (D.C.M.); viorela.popescu@defs.ase.ro (V.E.P.); andreea.voinea@defs.ase.ro (A.V.); 2 Sport and Health Department, Faculty of Medical and Behavioral Sciences, Constantin Brâncuși University of Târgu-Jiu, 210135 Târgu-Jiu, Romania; bogdan.niculescu@e-ucb.ro (B.N.); andreea.popescu@e-ucb.ro (A.P.); 3 Department of Physical Education and Sports, Faculty of Humanities, Valahia University of Târgoviște, 130105 Târgoviște, Romania; ancuta.pirvan@valahia.ro; 4 Doctoral School of Physical Education and Sports, State University of Physical Education and Sport, MD-2024 Chișinău, Moldova; cristina.pascan@mta.ro; 5 Faculty of Physical Education and Sports, National University of Physical Education and Sports, 060057 Bucharest, Romania; lucian.paun@unefs.ro

**Keywords:** exercise adaptation, recovery monitoring, overload, overreaching, biomarkers, multi-omics, metabolomics, cell-free DNA, circulating RNA, extracellular vesicles

## Abstract

Exercise adaptation and training maladaptation arise from overlapping metabolic, redox, inflammatory, endocrine, and tissue-remodeling processes, so the translational question is not whether biomarkers change but when, where, and for which decision they become informative. This narrative review develops a decision-linked framework for minimally invasive biomarkers across the recovery–overload continuum and treats biomarker meaning as a molecule–matrix–time–decision relationship rather than as a stand-alone peak. The framework is organized around five coupled layers: stimulus architecture, signaling and release biology, sampling matrix and pre-analytics, bout-relative kinetics, and the monitoring decision to be supported. Current evidence indicates that no single biomarker reliably separates productive remodeling from delayed recovery, tissue strain, non-functional overreaching, or early maladaptation. Classical chemistry remains useful for bounded tasks, especially delayed tissue strain and stress reactivity; cfDNA appears promising for rapid load sensitivity; targeted metabolite panels are strongest for recovery phenotyping; and circulating RNAs and extracellular-vesicle cargo add mechanistic depth but remain constrained by pre-analytical fragility and incomplete standardization. The central practical implication is that overload is better interpreted as progressive loss of signal resolution than as threshold-crossing and that sparse temporally staggered panels are more likely to aid monitoring decisions than isolated markers or untimed high-dimensional profiles. Progress will depend on purpose-specific panels, transparent analytical standards, and prospective validation against symptoms, performance, and established measures across sex, hormonal, circadian, and training contexts.

## 1. Introduction

Exercise adaptation begins as a controlled disturbance of homeostasis in which mechanical, metabolic, redox, immune, and endocrine signals are mobilized to drive remodeling across skeletal muscle and extra-muscular tissues [1,2,3]. Redox-responsive signaling and exercise-induced hormesis help explain why these perturbations can culminate in adaptation rather than net disruption [4,5,6,7,8,9]. The translational challenge is therefore not simply to document molecular movement but to determine how these signals resolve across recovery and when they become informative for monitoring decisions. Recent redox-centered syntheses further support the view that exercise-induced oxidative perturbation is most informative when interpreted as a context-dependent signaling layer shaping adaptation and recovery rather than as a uniformly deleterious by-product [10]. Current biomarker-based monitoring fails not because of a lack of measurable signals but because biomarker interpretation is systematically misaligned with signal kinetics, sampling context, and decision purpose.

That challenge is difficult because adaptation, delayed recovery, tissue strain, functional overreaching, and early maladaptation arise from overlapping biology rather than discrete pathways [11,12,13]. Consensus work on training load, injury risk, and illness risk supports viewing these states as a continuum rather than a binary threshold [14,15,16,17]. The same shifts in membrane disruption, inflammatory signaling, endocrine stress, or substrate turnover may reflect productive remodeling in one context and accumulating biological cost in another [18,19,20]. This overlap explains why isolated biomarkers rarely function as stand-alone classifiers of training status [21,22,23].

Recent multi-omics studies have sharpened mechanistic resolution by showing that exercise responses are distributed across tissues, timescales, and regulatory programs [24,25,26]. Human training studies, consortium protocols, and translational syntheses extend this signal across longer adaptation windows, multiple tissues, and applied monitoring contexts [27,28,29,30,31,32]. Yet most reviews still organize the field mainly by molecule class or platform, leaving the key translational gap unresolved: when does a marker become informative and in which matrix, at what bout-relative time, and for which monitoring decision [33,34,35,36,37,38,39]?

This article is intentionally a narrative review rather than a systematic review or meta-analysis. Its purpose is conceptual integration across heterogeneous biomarker classes, matrices, and sampling windows to support translational monitoring decisions, not exhaustive effect-size aggregation or formal certainty grading.

Accordingly, this review does not simply catalogue exercise-responsive molecules; it proposes a decision-linked interpretive framework for minimally invasive biomarkers across the recovery–overload continuum. Its distinctive claim is that biomarker utility should be judged at the level of a molecule–matrix–time–decision combination, not at the level of a molecule alone. The review is organized around three questions:

**Q1**. When does a biomarker become informative for a bounded monitoring decision rather than simply responsive to load?
**Q2**. Which matrix and bout-relative window make a given biomarker interpretable?
**Q3**. Do sparse, temporally layered panels provide more translational value than isolated biomarkers or untimed high-dimensional signatures?

Three propositions guide the review: (1) overload is better interpreted as progressive loss of signal resolution than as threshold crossing; (2) biomarker utility depends on temporal and mechanistic complementarity rather than platform novelty; (3) validation should be judged by improvement in bounded monitoring decisions rather than association with load alone. Although the primary emphasis is on athlete monitoring, the framework may also inform exercise medicine and rehabilitation, where repeated exercise exposure and incomplete recovery must be interpreted in a time-sensitive, context-dependent manner.

***Original Contribution of This Review*.** Unlike prior reviews that primarily catalogue exercise-responsive biomarkers by molecule class or analytical platform, the present work proposes a decision-linked interpretive framework in which biomarker utility is defined at the level of a molecule–matrix–time–decision combination. In practical terms, the review advances three linked claims: overload is better conceptualized as progressive loss of signal resolution than as threshold crossing; biomarker interpretation depends on temporal alignment with bounded monitoring decisions; and sparse, temporally layered panels are more likely to add translational value than isolated markers or untimed high-dimensional profiles. This review therefore aims not to expand the list of candidate biomarkers but to redefine the unit of interpretation in exercise biomarker research, shifting it from isolated molecular signals to temporally and contextually constrained decision units.

## 2. Interpreting Exercise Biomarkers: A Decision-Linked Systems Framework

Interpreting exercise biomarkers requires more than linking molecules to physiology; it requires linking them to stimulus architecture, release biology, sampling conditions, and the recovery environment in which adaptation unfolds. Because similar signals can accompany both productive remodeling and accumulating strain, interpretation depends on kinetics, matrix, and the bounded decision being supported. The framework proposed here is intended not only as a conceptual synthesis, but as an operational scaffold for sampling design, panel construction, and prospective validation. Unlike platform-centered reviews that mainly ask whether a molecule is exercise responsive, this framework asks when a marker becomes interpretable, in which matrix, and for which decision. The literature synthesis prioritized human repeated-measures studies with clear bout-relative timing, defined sampling matrices, and direct translational relevance to athlete monitoring; details are provided in Appendix A.

The analytical premise of this review is that a measured biomarker reflects a transformation chain between exercise dose and circulating readout. Mechanical tension, calcium flux, energetic strain, hypoxia, catecholamine signaling, glucocorticoid tone, and immune trafficking converge on nodes such as AMPK, PGC-1α, mTORC1, NF-κB, and Nrf2 [1,2,3]. These signaling networks shape downstream remodeling outcomes such as substrate switching and mitochondrial adaptation [4,5,6], while broader redox-responsive and hormetic signaling influences how those responses are resolved [7,8,9]. This time-resolved systems perspective is also consistent with recent models proposing that repeated training adaptation depends on preserving temporal separability between energy-sensing and anabolic programs rather than allowing persistent energetic congestion to accumulate [40].

A second interpretive step is matrix conversion. Biomolecules may enter plasma directly or circulate within protein complexes and extracellular vesicles [41]. They may later appear in saliva or urine [35,36] with different delays and analytical liabilities [42,43,44]. The same training bout can therefore generate substantially different readouts depending on matrix, collection device, and processing delay [37,38,39]. Interpretation also shifts according to whether the goal is same-day load quantification [45,46,47,48] or later mechanistic phenotyping [49,50,51].

**Figure 1:**
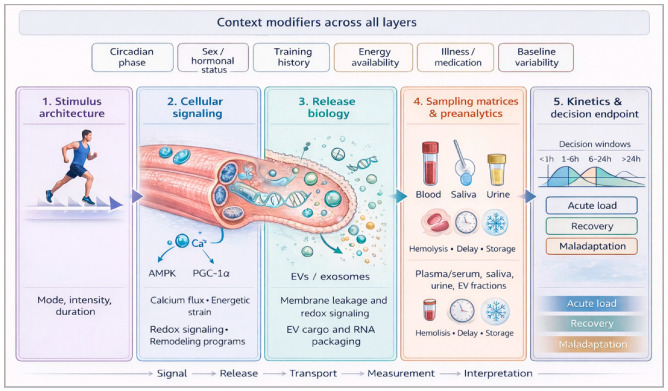
Decision-linked systems framework for translational exercise biomarker interpretation. The schematic links whole-body exercise exposure to a muscle-cell signaling layer, release routes (e.g., membrane leakage, cfDNA shedding, and EV/RNA packaging), minimally invasive sampling matrices, and bounded monitoring decisions. The left-to-right flow emphasizes movement from exercise dose to interpreted decision, whereas the anatomical and cellular visuals make explicit the biological steps that intervene between training load and measurable signal. Interpretability emerges from the alignment of biomarker kinetics, sampling matrix, and decision endpoint rather than from the magnitude of any single signal. Contextual modifiers such as circadian phase, sex or hormonal status, training history, energy availability, illness, medication, and baseline variability act across all layers. Conceptual synthesis based on the exercise signaling and adaptation literature, translational athlete-monitoring frameworks, and matrix/pre-analytical considerations [1,2,3,21,22,35,36,37,38,39,41].

Throughout this manuscript, biomarker families are interpreted through five coupled layers: stimulus architecture, signaling and release biology, sampling matrix and pre-analytics, biomarker-domain kinetics, and the decision endpoint to be informed. This framework (Figure 1) is used to compare biomarkers against bounded monitoring tasks such as acute load sensitivity, delayed tissue strain, recovery sufficiency, and early maladaptation rather than against the unrealistic expectation of universal diagnostic thresholds. In that sense, the main original contribution of the review is operational rather than taxonomic: it shifts the unit of interpretation from the isolated molecule to the matched signal-context package.

Operationally, a biomarker is treated as decision-informative only when three conditions are met simultaneously: its kinetics align with the intended sampling window, its matrix permits interpretable measurement under known pre-analytical constraints, and its signal maps onto a predefined monitoring decision rather than generic exercise responsiveness. This operational definition is used throughout the manuscript to distinguish mechanistic interest from translational readiness.

From a practical perspective, the proposed framework can be operationalized by matching marker choice and sampling time to a prespecified decision rather than to platform availability alone. Figure 1 therefore visualizes the full chain from the whole-body exercise dose to muscle-cell signaling, release pathways, minimally invasive sampling, and bounded monitoring interpretation. When the monitoring objective is acute training-load quantification, early, rapidly responsive markers such as cfDNA or selected salivary stress signals are most informative. For delayed tissue-strain or recovery assessment, markers with slower kinetics, such as CK or targeted metabolite profiles, are better prioritized within 24–48 h windows. When the objective is to interpret recovery status or emerging maladaptation, persistent multi-domain patterns integrating kinetics, symptomatology, and performance become more informative than isolated signals. The framework is therefore intended to guide purpose-specific marker selection and sampling logic rather than to define universal biomarker thresholds.

### Common Translational Criteria Used Across Biomarker Families

Across subsequent sections, biomarker families are compared using the same appraisal lens: Which biological layer is being sampled? How rapidly does the signal rise and resolve? Which matrix best captures it under realistic field conditions? Which pre-analytical liabilities most threaten interpretation? And which bounded decision, if any, does the biomarker improve beyond established measures? Applying this common lens helps prevent platform-driven description and keeps the review anchored to practical inference rather than molecular novelty alone.

Terms such as moderate maturity, emerging maturity, or limited readiness are therefore used comparatively rather than diagnostically. A biomarker may be useful for one narrow task, such as same-day load sensitivity, yet weak for delayed recovery classification or early maladaptation screening. Making that distinction explicit is essential for fair comparison across classical chemistry, metabolite panels, cfDNA, circulating RNAs, and extracellular-vesicle cargo.

## 3. Recovery–Overload Continuum as Progressive Loss of Signal Resolution

Adaptation and maladaptation are best interpreted as positions along a continuum running from resolved perturbation to delayed recovery, functional overreaching, non-functional overreaching, and overtraining-like maladaptation. Across these states, the underlying biology—metabolic strain, redox signaling, immune activation, endocrine stress, and tissue remodeling—overlaps substantially [11,16,17]. Recent syntheses in resistance exercise and broader overtraining contexts support the same interpretation [52,53]. What changes most is not pathway identity but the timing, persistence, and coordination of signals.

This shared biology makes isolated biomarkers intrinsically difficult to interpret. A rise in CK, cfDNA, cytokines, or redox-sensitive metabolites may reflect useful remodeling after a novel bout, transient delayed recovery, or accumulating tissue cost [54,55,56]. The repeated-bout effect deepens the ambiguity because prior exposure can blunt membrane leakage while preserving transcriptional and cellular adaptation [57,58]. Lower amplitude therefore does not necessarily imply lower biological work.

Operationally, the shift toward overload is better conceptualized as progressive loss of signal resolution rather than as accumulation of a single analyte. In a resolved state, fast load-sensitive signals rise and normalize on schedule, delayed tissue-strain markers show limited spillover, and cross-domain coupling remains low. As recovery deteriorates, signals emerge at the wrong time, persist beyond their expected window, and become increasingly concordant with symptoms, performance decrement, and repeated-session carryover. The most informative warning sign is therefore not an isolated peak but the persistence, mistiming, and cross-domain coherence of multiple signals across successive windows [18,19,20]. Earlier overtraining literature anticipated this interpretive problem [12,13], and recent systematic review evidence on team athletes reaches a similar conclusion [23]. 

**Figure 2:**
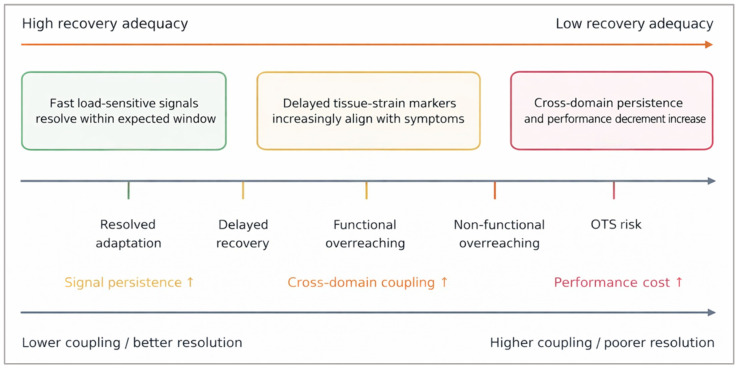
Axis-based representation of the recovery–overload continuum as progressive loss of signal resolution. The schematic is conceptual rather than diagnostic and positions states from resolved perturbation and productive recovery on the left toward delayed recovery, functional overreaching, non-functional overreaching, and overtraining-like maladaptation on the right. The upper arrow indicates declining recovery adequacy from left to right, whereas the lower arrow indicates increasing cross-domain coupling, signal persistence, symptom concordance, and performance cost across the continuum. Conceptual synthesis based on the recovery–load continuum, overtraining, and athlete-monitoring literature [11,12,13,14,15,16,17,18,19,20,23,52,53].

As illustrated in Figure 2, overload is conceptualized as a progressive loss of signal resolution, characterized by mistimed, persistent, and increasingly concordant biomarker responses.

## 4. Interpretive Limits of Single Biomarkers in Exercise Monitoring

If Section 3 explains why biology along the recovery–overload continuum remains intrinsically overlapping, the operational consequence is straightforward: single-marker monitoring fails for predictable reasons. Recent workload-monitoring syntheses continue to highlight CK, lactate dehydrogenase, myoglobin, cortisol, testosterone, the testosterone:cortisol ratio, C-reactive protein, and selected cytokines because they are easy to collect and easy to explain [21,23]. The earlier sports medicine literature on CK and related metabolic markers helps explain why this appeal persists despite limited interpretive specificity [59,60].

CK remains the clearest example. It is widely used as a proxy for muscle damage, yet its concentrations are strongly influenced by sex, muscle mass, ethnicity, training history, eccentric load, and sampling time. Some athletes show large post-exercise increases with little functional impairment, whereas others demonstrate meaningful performance decrements with comparatively modest CK responses [54,57,58]. Methodological reviews on CK monitoring underscore the same problem [59,60].

Endocrine and immune markers have parallel limitations. Cortisol reflects training load but also sleep restriction, caloric stress, and circadian phase [61,62]. Salivary alpha-amylase is sensitive to autonomic state yet also to sampling method and oral conditions [63,64]. Cytokines and leukocyte shifts may capture important biology, but their meaning is inseparable from exercise modality, tissue damage burden, recovery timing, and the broader nutritional context in which stress reactivity is interpreted [65,66,67].

Population thresholds are therefore seductive and usually blunt. A value that appears abnormal in one athlete may be entirely ordinary in another, whereas a modest within-athlete deviation can be meaningful if it appears at the wrong time, in the wrong matrix, and alongside the wrong companion signals. Most apparent contradictions in the single-marker literature can be traced to one of three problems: biological dispersion between athletes, mismatch between marker kinetics and the sampling window, or interpretive drift in which a stress-reactive signal is asked to classify tissue strain or adaptation status. The operational unit of interpretation should therefore be the athlete in context [18,19,20], not the molecule in isolation or a threshold borrowed from a different population or sampling routine [21,23]. This limitation can be expressed operationally as follows:
**Why single biomarkers fail: a structural limitation**Single biomarkers fail not because they are uninformative, but because their meaning is inherently context-dependent.▪The same signal has a different meaning across time windows;▪The same value has a different meaning across matrices;▪The same response has a different meaning across athletes.Therefore, biomarker interpretation without temporal and contextual alignment is structurally invalid.

## 5. Sampling Matrices and Pre-Analytical Constraints

A biomarker does not mean the same thing in every fluid. Different matrices capture different biological layers, impose different pre-analytical constraints, and permit different sampling frequencies [35,36]. Matrix choice should therefore be treated as part of study design rather than as a late operational convenience [21].

### 5.1. Blood and Plasma

Blood and plasma remain the most information-dense matrices for exercise monitoring [21,22]. They support established clinical chemistry, endocrine assays, cytokines, untargeted and targeted metabolomics, cfDNA, circulating RNA analyses, and EV isolation [25,28,36]. This richness makes blood the default choice when mechanistic breadth matters more than sampling burden.

The cost of this richness is analytical fragility. Posture, tourniquet time, hemolysis, centrifugation delay, freeze–thaw history, and even the type of collection tube can distort interpretation [37,68,69]. Emerging markers such as cfDNA and EV cargo are especially vulnerable to these pre-analytical choices [38,39], which means analytical sophistication cannot compensate for poor specimen handling.

### 5.2. Urine

Urine is attractive because it is non-invasive, repeatable, and already operational in many athletic environments [35,36]. It is particularly well suited to metabolomic profiling because it contains rich downstream biochemical information [42,43,44]. Exercise-metabolomics studies and reviews reinforce this practical advantage [70,71].

Its limitations are equally important. Hydration, urine concentration, diet, time of day, and recent exertion can all influence the measured profile [35,42,43,44]. Recent rowing studies show both the promise and the caution required [72,73]. Urinary metabolomics can reveal exercise-responsive signatures associated with tissue strain and metabolic stress, but those signatures remain context sensitive and not yet ready for universal cut-points [74,75].

### 5.3. Saliva

Saliva offers perhaps the most practical route to high-frequency, low-burden monitoring [35,36]. Salivary cortisol and testosterone have long been used in sport and stress research [62,63]. Because salivary stress-sensitive signals are strongly context dependent, their interpretation should be aligned with energy availability, time of day, and recent intake rather than read in isolation [35,36,62,63]. Alpha-amylase and IgA extend this matrix toward autonomic and mucosal immune monitoring [64,76].

However, salivary readouts are exquisitely sensitive to flow rate, oral health, recent food intake, hydration status, and assay method [35,36]. Saliva is therefore often most useful as part of a layered panel—particularly for rapid stress-sensitive or mucosal measures—rather than as a stand-alone substitute for blood chemistry [66].

### 5.4. Extracellular Vesicle-Enriched and Hybrid Sampling Strategies

From a translational perspective, EVs are promising but not yet mature. Isolation strategy, pre-clearing steps, storage conditions, and characterization criteria can all change what the investigator believes the biology says [38,39]. Until reporting discipline and inter-laboratory comparability improve, EV-enriched fractions should be treated primarily as mechanistic enrichment tools rather than as routine field-ready biomarkers [41].

Taken together, sampling matrices do not merely represent alternative collection media but distinct biological filters that determine which layer of the exercise response becomes visible. Blood provides the highest analytical breadth but imposes strict pre-analytical discipline, whereas urine and saliva offer greater feasibility for repeated field monitoring at the cost of additional contextual variability. Consequently, matrix selection should be treated as a design decision aligned with the biological layer of interest, the required sampling frequency, and the practical constraints of the monitoring environment.

## 6. Established Biomarker Domains: Utility and Interpretive Boundaries

The expansion of omics platforms should not produce a false dichotomy between classical and modern biomarkers. Recent translational reviews have argued persuasively that established chemistry and hematological indices remain clinically interpretable, inexpensive, and operationally accessible [21,22,23]. The earlier sports medicine literature reached similar conclusions for CK and related metabolic markers [59,60].

Muscle-damage-associated markers, such as CK, lactate dehydrogenase, and myoglobin, are best understood as indicators of membrane disruption and tissue strain rather than as direct readouts of adaptation. Their value increases in eccentric, novel, or high-mechanical-load settings [54,57,58], particularly when aligned with symptoms, force loss, or objective performance decrement [55,56]. Related hypertrophy-focused evidence likewise reinforces that structural adaptation cannot be inferred from damage-associated chemistry alone because anabolic remodeling depends on nutritional and signaling context not captured by CK-type leakage markers [77].

Endocrine and immune variables occupy a different interpretive domain. Cortisol, testosterone, selected cytokines, leukocyte subsets, and mucosal immune markers can inform stress load, readiness, and recovery sufficiency [61,63]. Cytokine and leukocyte changes add an immune context [65,66,67]. These variables become most useful when interpreted longitudinally against individualized baselines, standardized sampling routines, and the accompanying training and recovery context [18,19,20].

Accordingly, classical biomarkers retain translational value when they answer restricted questions such as whether tissue strain has accumulated, whether recovery remains incomplete, or whether a stress-sensitive signal has shifted beyond the athlete’s usual range [21,23]. They lose value when they are treated as omnibus classifiers of adaptation, recovery, and overload simultaneously [18,19,20].

## 7. Emerging Omics-Derived Biomarker Families

The promise of omics is not numerical scale, per se, but layer-specific biological resolution across release mechanisms and time. Within the present framework, the major omics families address different ambiguities: targeted metabolites are most useful for biochemical state discrimination in early-to-intermediate or delayed windows; cfDNA for rapid same-day biological cost; circulating RNAs for regulatory interpretation when classical chemistry or metabolite signals are discordant; and extracellular-vesicle cargo for mechanistic enrichment of inter-tissue communication. Translational maturity, however, remains uneven. At present, targeted metabolite panels and cfDNA offer the clearest restricted use cases, whereas circulating RNAs and extracellular-vesicle cargo are better treated as adjunct mechanistic layers than as routine monitoring tools.

### 7.1. Metabolites and Metabolic Fingerprints

Metabolomics is especially attractive because exercise rapidly reorganizes substrate use, redox handling, amino acid flux, lipid turnover, and purine metabolism [44,70,74,75]. Current translational evidence is strongest for targeted or semi-targeted repeated-measures panels designed to answer bounded questions about acute load, substrate stress, or delayed recovery [72,73]. By contrast, untargeted datasets are primarily discovery-generating and can become unstable when fasting state, hydration, sex, or exercise timing are incompletely controlled [69,71,78]. Annotation confidence, normalization strategy, and batch handling add further instability [79,80,81]. Annotation resources such as HMDB and reference metabolome datasets improve interpretability [43,82], but they cannot compensate for weak sampling logic or poor analytical discipline. Accordingly, targeted panels are closer to practice, whereas untargeted workflows still require external validation before operational use.

### 7.2. Circulating Cell-Free DNA and Fragmentomics

cfDNA is one of the most compelling emerging biomarkers in exercise physiology because its temporal behavior is unusually rapid. Intense or exhaustive exercise produces a sharp rise in circulating cfDNA that often peaks immediately or shortly after the bout and declines far sooner than CK or C-reactive protein [45,46,47,48]. Exercise-associated cfDNA probably reflects a mixture of neutrophil extracellular trap formation, apoptosis, necrosis, and possibly mitochondrial DNA release [49,50,51], which helps explain both its acute load sensitivity and its dependence on sampling conditions.

These kinetics make cfDNA particularly attractive as a fast load-sensitive signal. Within the present framework, its likely value is not to replace tissue-strain markers, but to solve a different interpretive problem: capturing the immediate biological cost of a session before delayed markers become informative. At the same time, lessons from liquid-biopsy science underscore that concentration is only one layer of meaning [49,50,51]. Fragment size and tissue origin materially influence interpretation [48,49,50,51], while sample handling and processing delays add a second layer of pre-analytical noise [37]. Compared with metabolite panels, cfDNA often offers cleaner temporal contrast but weaker stand-alone tissue attribution, which is why its strongest near-term use case is as the fast layer within a staggered multi-marker panel rather than as a delayed-recovery classifier.

### 7.3. Circulating microRNAs and Long Non-Coding RNAs

Circulating microRNAs and long non-coding RNAs are attractive because they may provide access to regulatory biology that classical leakage markers only approximate. Acute exhaustive exercise and endurance training modify selected circulating miRNAs [83,84,85], and sport-specific training-cycle studies suggest additional sensitivity in athletes [86,87]. Emerging work on lncRNAs adds a further regulatory layer [88,89].

Yet current translational maturity remains limited by candidate-specific reproducibility, normalization strategy, small-cohort discovery designs, and heterogeneous timing. At present, circulating RNAs are best interpreted as regulatory enrichments layered onto classical chemistry or metabolite data rather than as stand-alone monitoring solutions. Their near-term translational role is therefore more likely adjudicative than screening-oriented: most useful when layered onto discordant classical or metabolic profiles to explain why a response pattern emerged, not when asked to classify overload on their own.

### 7.4. Extracellular Vesicle Cargo

If circulating RNAs represent one regulatory layer, extracellular vesicle cargo offers a multiplexed extension of the same logic. Exercise rapidly increases circulating vesicle release [90], and these particles can transport proteins, lipids, metabolites, and nucleic acids involved in inter-tissue communication and systemic adaptation [41].

The translational limitation is standardization. Extracellular-vesicle biology is mechanistically rich, but isolation strategy, pre-clearing steps, storage conditions, characterization quality, and cargo quantification still vary enough to constrain inter-study comparability [38,39]. At present, EV-based approaches are best viewed as discovery-enabling and mechanism-refining tools rather than routine stand-alone monitoring platforms [41]. This makes the EV cargo conceptually attractive for studying multi-tissue adaptation, yet currently, it is less robust than cfDNA or targeted metabolites for routine field deployment, first-line monitoring, or prospective panel validation.

### 7.5. Integrative Multi-Omics Models

From a translational perspective, maximal dimensionality is rarely the goal. The principal value of multi-omics lies in identifying smaller sets of biologically complementary markers with clearer kinetics and stronger interpretive specificity. Multi-omics should therefore function as a reduction strategy, not an end-state platform: it should discover combinations of fast load-sensitive, delayed tissue-strain, and regulatory signals that add non-redundant information beyond classical monitoring. Current evidence thus supports the greatest operational maturity for bounded use of classical chemistry, targeted metabolite panels, and cfDNA [30,31], with lower maturity for circulating RNAs and extracellular-vesicle cargo until reproducibility, pre-analytical standardization, and prospective validation improve [79,81].

The most credible multi-omics models will be those that move deliberately from discovery space to operational space: first identify candidate clusters; then reduce them to sparse, kinetically complementary panels; and finally test whether those reduced panels improve interpretation relative to simpler baselines. Under that standard, complexity is justified only when it clarifies decisions, not when it merely expands datasets. 

Table 1 summarizes candidate biomarker families across the recovery–overload continuum, including their typical time scales, interpretive value, and practical limitations.

## 8. Temporal Architecture and Bout-Relative Sampling Windows

In exercise biomarker research, timing is part of the signal rather than ancillary metadata. A sample described only as post-exercise is biologically ambiguous because it may reflect immediate sympathetic activation and cfDNA release [45,48], intermediate metabolic redistribution [25,72], or later membrane leakage and recovery biology [54,83].

Different biomarker families therefore occupy partially distinct temporal windows. cfDNA and selected salivary stress-sensitive signals rise early and resolve quickly [47,48]. Metabolite fingerprints often dominate the early-to-intermediate window [72]. Delayed tissue-strain markers such as CK become more informative later [54], whereas regulatory layers such as circulating RNAs or extracellular-vesicle cargo may extend interpretive resolution into subsequent recovery phases [86,90]. Untimed sampling effectively collapses biologically distinct signals into apparent noise, which explains much of the inconsistency observed across the exercise biomarker literature. Repeated, purpose-specific sampling usually yields more biological information than a single untimed draw.

**Figure 3:**
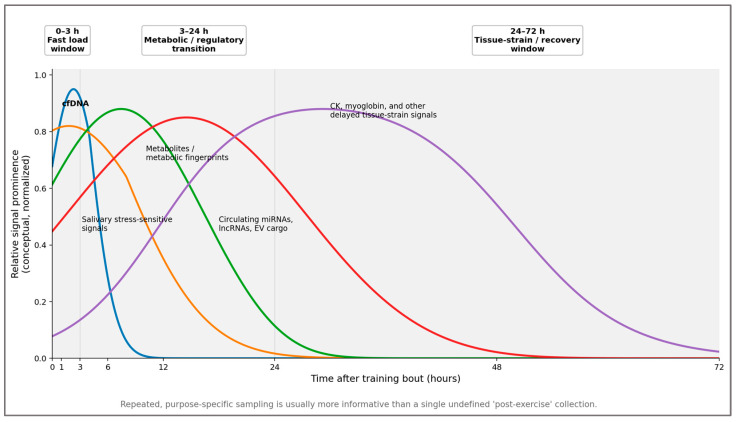
Approximate kinetic windows of major biomarker families after a training bout. The figure highlights representative early (0–3 h), intermediate (3–24 h), and delayed (24–72 h) windows for major biomarker domains. Blue denotes cfDNA, orange salivary stress-sensitive signals, green metabolite fingerprints, red circulating RNAs/EV cargo, and purple delayed tissue-strain markers such as CK or myoglobin. The *y*-axis represents relative signal prominence normalized conceptually across domains rather than absolute concentration, and the window labels at the top indicate approximate periods of dominant interpretive utility. The curves are conceptual rather than universal and should be interpreted relative to stimulus novelty, eccentric load, and chronic training history, which can shift both the amplitude and temporal positioning of biomarker responses. Approximate kinetic windows synthesized from the representative literature on cfDNA, salivary stress markers, metabolomic responses, circulating RNAs and extracellular vesicles, delayed tissue-strain markers, circadian timing, and repeated-bout effects [45,46,47,48,54,57,58,72,83,86,90,91,92].

Chronobiology adds a second temporal structure that is often underestimated. Time of day influences exercise capacity, muscle metabolism, mitochondrial function, hormonal background, and recovery kinetics [34,91,92], so the same numerical value may not carry the same biological meaning in morning and late-day samples. Exercise-timing and circadian studies therefore support the explicit control, or at least transparent reporting, of clock time in biomarker research. Figure 3 schematizes these partially overlapping bout-relative kinetic windows.

**Figure 4:**
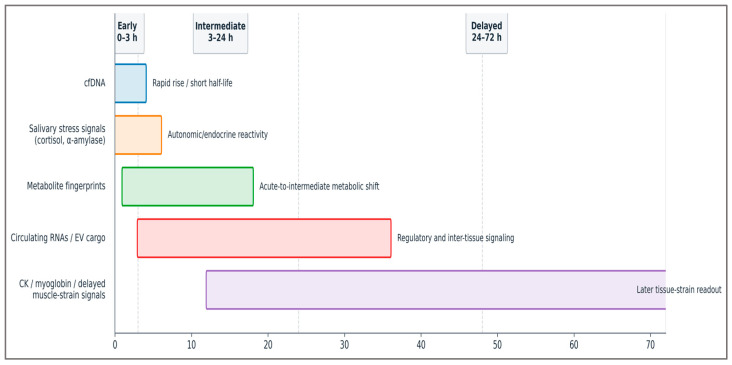
Complementary sampling-oriented timeline of representative biomarker families after a training bout. In contrast to the conceptual overlap curves in Figure 3, this schematic restates the same kinetic logic as an operational view of when different biomarker families are most informative for monitoring across early (0–3 h), intermediate (3–24 h), and delayed (24–72 h) windows. Horizontal bar length indicates approximate periods of greatest decision utility, not fixed analytical cut-points, and the color coding matches Figure 3 (blue: cfDNA, orange: salivary stress signals, green: metabolites, red: circulating RNAs/EV cargo, purple: delayed tissue-strain markers). The windows are illustrative rather than universal and should be individualized according to training history, stimulus familiarity, and recovery context. Operational sampling-oriented timeline derived from the same biomarker-domain kinetic literature summarized in Figure 3 [45,46,47,48,54,57,58,72,83,86,90,91,92].

To complement the kinetic-overlap view shown above, Figure 4 translates the same temporal logic into a simplified, sampling-oriented timeline that highlights when major biomarker families are most informative in practice.

Chronic training history can shift these windows rather than merely rescale their amplitude. Repeated exposure to a familiar stimulus can blunt membrane leakage and CK spillover through the repeated-bout effect and may shorten the apparent delayed-recovery window, whereas novel or eccentric loading can broaden or right-shift delayed tissue-strain signals and prolong carryover into the next session [54,57,58]. For that reason, Figure 3 and Figure 4 should be read as individualized movable windows around a training history, not as fixed universal templates. More broadly, oscillatory load structure may also reshape the epigenetic background against which these kinetic windows are expressed, suggesting that chronic training history can modify not only amplitude but also the interpretive spacing between biological windows [93].

## 9. Sources of Biological and Methodological Heterogeneity

Biomarkers fail in practice not only because the biology is complex but because context is routinely stripped away before interpretation begins. Sex, menstrual status, and training history shape biomarker responses [33,94]. Nutritional state, low energy availability, and broader endocrine context add further biological variation [33,94,95,96,97]. Training–fuel coupling frameworks likewise support treating substrate timing and energy availability as interpretive covariates rather than residual noise when repeated biomarker measurements are compared across sessions [98].

A related challenge is transferability. Panels derived in tightly controlled male or single-sport cohorts may lose performance when moved to different training modes, age ranges, recovery environments, or biologically characterized female cohorts [33,94,96]. The longstanding under-representation of women in sports and exercise medicine research makes this transfer problem especially persistent [99,100]. External validity in this field should therefore be treated as a mechanistic test, not a statistical courtesy: if the same panel changes meaning across contexts, the problem is not only calibration but biological incompleteness.

This issue is especially consequential for women. Menstrual phase, hormonal milieu, contraceptive status, and energy availability are not nuisance variables to be adjusted away post hoc [33,94]. Emerging redox and proteomic data in female athletes show that these factors can alter phenotype at the level of mechanism [96,97].

High-dimensional platforms amplify these vulnerabilities. Omics technologies can detect patterns invisible to conventional assays, but they are unusually sensitive to batch effects, incomplete metadata, unstable normalization, and underpowered discovery cohorts [68,69,79]. Multiple testing artifacts and weak statistical control can further distort interpretation [78,79,80,81]. In this setting, pre-analytics and statistics are inseparable from biological inference.

## 10. Design Principles for Decision-Linked Multi-Marker Panels

In practice, panels should be assembled according to four design rules: temporal complementarity, mechanistic non-redundancy, matrix feasibility, and decision specificity. Temporal complementarity prevents all markers from sampling the same biological window; mechanistic non-redundancy reduces the risk of repeatedly measuring the same process; matrix feasibility determines whether the panel can be implemented at the required sampling frequency; and decision specificity forces the investigator to define in advance what action a given pattern should trigger. Without these constraints, panel design becomes an exercise in accumulation rather than inference [21,22]. Recent multi-omics-to-personalized-training frameworks make the same point from a systems perspective [32]. A translational panel should therefore be judged not by how many analytes it contains but by whether it improves a bounded monitoring decision.

Panel construction should begin with the decision to be supported rather than with the platform available. Acute load quantification, delayed recovery surveillance, tissue-strain detection, mechanistic phenotyping, and early maladaptation screening are distinct inferential tasks [21,22]. Personalized multi-omics frameworks likewise argue against collapsing them into a single biomarker solution [32]. A useful panel is therefore complementary rather than maximal: each component should represent a different biological layer, occupy a deliberate time window and reduce uncertainty that would otherwise remain unresolved. The validation target should be specified at the same stage as panel construction.

A practical panel usually requires at least three interpretive layers. The first is a rapidly responsive load-sensitive layer, such as cfDNA or a selected salivary stress marker [48], sampled immediately or within the first few hours after a bout. The second is a delayed tissue-strain layer, such as CK, myoglobin, or a targeted metabolite pattern linked to muscle damage [72], sampled 24–48 h later. The third is a contextual or regulatory layer consisting of circulating RNAs, extracellular-vesicle cargo, or athlete-specific metadata [86,88,90] that helps explain why a given response pattern emerged and whether it is resolving as expected. Panels become translationally stronger when the chosen markers are mechanistically non-redundant, temporally staggered, explicitly tied to a predefined decision, and sparse enough to remain implementable under real monitoring conditions.

Matrix selection should follow the same logic. Blood is preferable when mechanistic breadth or assay flexibility is required; saliva when repeated low-burden sampling is operationally decisive; and urine when metabolic fingerprinting and recovery surveillance are the primary aims. The goal is not maximal information density in the abstract but complementarity between the matrix, kinetics, mechanistic layer, and decision domain. Table 2 translates this logic into practical panel examples, suggested sampling windows, and intended operational interpretations.

A concrete example is a 72 h team-sport monitoring workflow anchored to within-athlete baselines. A minimal operational panel could include plasma cfDNA and salivary cortisol or alpha-amylase collected immediately post-session, CK plus a targeted metabolite panel collected at 24 h, and soreness together with a simple neuromuscular test such as countermovement jump at 24 and 48 h. The intended inference is not generic fatigue but whether load cost appears to be resolving on schedule or whether cross-domain persistence suggests delayed recovery. Operationally, the logic can be reduced to a simple sequence: when the fast post-bout layer rises as expected, but delayed biochemical, symptom, and performance signals normalize within their anticipated window, the session is most consistent with productive loading; when the fast layer remains elevated at the next exposure or delayed tissue-strain markers stay high alongside soreness or force loss, interpretation should shift toward incomplete recovery; when that cross-domain persistence recurs across successive sessions or microcycles, concern should escalate toward early maladaptation and trigger review of workload and recovery context rather than reliance on any single threshold.

### Testable Predictions and Minimal Validation Pathway

The framework advanced in this review yields four explicit, testable predictions:

**P1**. Temporally discordant profiles should outperform absolute peak magnitude: an early load-sensitive signal that remains elevated into the next exposure, or a delayed tissue-strain marker that appears outside its expected window, should carry greater concern than an isolated peak within its expected window.
**P2**. Cross-domain coupling should increase as recovery adequacy declines: when fast signals, delayed tissue-strain markers, symptoms, and performance decrement begin to move together across repeated sessions, the probability of delayed recovery or emerging maladaptation should rise.
**P3**. Sparse temporally staggered panels should outperform untimed or mechanistically redundant panels, because added variables should improve inference only when they sample different biological layers.
**P4**. Within-athlete trajectories should outperform population thresholds for repeated monitoring tasks, especially when sex, hormonal milieu, circadian timing, and training history are modeled explicitly rather than treated as residual noise.

These predictions imply a minimal prospective validation design. At minimum, athletes should be followed longitudinally across repeated sessions or microcycles with one fast window (immediately post-bout or 1–3 h), one delayed window (24–48 h, with optional 72 h follow-up), and a predefined decision endpoint such as same-day load classification, delayed recovery detection, tissue-strain surveillance, or early maladaptation flagging. Interpretation should be blinded or rule-based, benchmarked against symptoms, simple performance measures, and established clinical chemistry, and then externally replicated in an independent cohort. A result would support the framework if temporally matched, sparse, decision-specific panels improve these bounded judgments more than single markers or untimed high-dimensional profiles. Conversely, the framework would be weakened if untimed or mechanistically redundant panels perform equally well across contexts, or if population thresholds consistently outperform within-athlete trajectories under repeated monitoring conditions.

## 11. Validation Requirements and Current Limitations

### 11.1. Validation and Reporting Standards

Validation is the point at which many attractive biomarker claims fail translational scrutiny. Discovery alone is insufficient. Future studies should prioritize repeated-measures longitudinal designs, explicit characterization of the exercise stimulus, justified bout-relative sampling windows, transparent matrix handling, and normalization strategies that match the biology under study [21,22]. Human exercise-omics consortia have also shown the value of dense, protocol-defined sampling and standardized metadata [28,29]. A candidate panel should do more than correlate with load; it should improve interpretation relative to established measures, remain stable under realistic pre-analytics, and demonstrate external validity across cohorts, training modes, and recovery contexts. The decisive question is not whether a panel is statistically associated with load but whether it improves a bounded action or judgment.

A useful translational validation pathway can be viewed as four sequential tests. First, analytical validity asks whether the marker can be measured reproducibly under realistic pre-analytical conditions. Second, kinetic validity asks whether the signal behaves consistently within the intended bout-relative sampling window. Third, contextual validity asks whether interpretation remains stable across sex, hormonal milieu, training mode, circadian timing, nutritional state, and recovery environment. Fourth, decision validity asks whether the marker or panel improves an actionable judgment beyond symptoms, performance testing, and established chemistry. Many candidate biomarkers appear promising at one stage yet remain unproven at the next, which is one reason discovery enthusiasm often outpaces implementation. A panel that fails any earlier step should not be advanced to routine monitoring simply because its multivariate classification performance looks attractive in a small cohort.

Validation must also be analytically domain-specific. Circulating RNA studies should meet MIQE-consistent expectations [68]. Metabolomics studies should document sample handling and signal identification [69,78], as well as batch control, missing-data strategy, and multiple-testing procedures [79,80,81]. Extracellular vesicle studies should align with MISEV [39], and all high-dimensional workflows should distinguish clearly between discovery, internal validation, external confirmation, and downstream decision utility. These domain-specific standards should be paired with sport- and exercise-specific reporting disciplines that explicitly define the exercise stimulus, recovery interval, time of day, participant sex or hormonal status, and repeated-measures context [14,15,20,33]. Predefined analysis plans, transparent feature selection, and careful treatment of missingness are especially important in settings where the ratio of variables to participants is unfavorable. Relevant paired guidance therefore includes consensus frameworks on sport load and recovery together with methodological standards for participant characterization in women athletes [14,15,20,33].

What the field requires is not an ever-expanding inventory of candidate molecules but a smaller number of well-justified panels with known kinetics, explicit pre-analytical boundaries, mechanistic anchors, and clearly defined decision domains. A translational panel should answer a bounded question, specify its expected kinetic window, tolerate realistic pre-analytics, and show that it changes interpretation or action rather than simply adding molecular detail.

In practical terms, prospective validation of a decision-linked framework should move beyond cross-sectional associations and test whether rule-based, purpose-specific panels improve bounded monitoring decisions over time. A minimally adequate design should combine within-athlete baselines, at least one fast and one delayed sampling window, predefined decision end points, blinded or rule-based interpretation, and external replication in an independent cohort. Validation should then ask whether the selected panel improves inference relative to symptoms, performance measures, and established clinical chemistry, and whether that added value remains stable across training modes, sexes, hormonal contexts, and recovery environments.

Table 3 summarizes these analytical, contextual, and validation requirements in a practical reporting framework for translational exercise biomarker studies.

### 11.2. Current Limitations of the Evidence Base

This review is narrative rather than systematic and does not attempt a formal meta-analysis. That choice fits the aim of building an interpretive framework across highly heterogeneous studies, but it limits quantitative comparison across biomarkers, matrices, populations, and sampling windows. The review also prioritizes minimally invasive markers and field-relevant monitoring questions, so biopsy-derived signals, imaging outcomes, and highly specialized laboratory approaches are discussed only insofar as they inform translational interpretation. No formal risk-of-bias instrument or certainty-grading scheme was applied; accordingly, judgments about translational maturity should be read as interpretive syntheses of mechanistic, analytical, and practical considerations rather than as evidence-certainty ratings. Within this context, the proposed framework should be interpreted as a decision-guiding model that now requires prospective empirical validation in athlete monitoring, exercise medicine, and rehabilitation settings.

A second limitation is the evidence base itself. Many candidate omics-derived signals remain supported by small, protocol-specific cohorts with incomplete external validation, and their meaning may shift across sex and hormonal milieu, sport type, circadian timing, energy availability, and recovery environment. These constraints reinforce the central argument of this review: decision-grade panels must be validated longitudinally against practical end points before routine adoption, and RNA- or EV-based layers should currently be interpreted as adjunctive rather than definitive.

## 12. Conclusions and Research Priorities

Exercise adaptation and training maladaptation differ less owing to the presence of any single molecular signal than to the timing, persistence, coordination, and context of overlapping metabolic, redox, immune, endocrine, and tissue-remodeling responses. Biomarker meaning is therefore not a property of the molecule alone but of the molecule in the right matrix, at the right time, for the right decision.

The field does not primarily need more exercise-responsive molecules; it needs fewer, better-timed, better-validated decisions. The most defensible translational path is purpose-specific multi-marker monitoring anchored to explicit sampling windows, within-athlete baselines, and prespecified actions. Classical chemistry, targeted metabolite panels, cfDNA, and selected regulatory signals should be treated as complementary layers whose value depends on kinetic fit, pre-analytical discipline, and added decision utility beyond symptoms, performance, and established measures across sex, hormonal milieu, circadian timing, training history, and recovery context. Near-term progress will depend on sparse panels, transparent decision rules, and prospective longitudinal validation rather than on ever larger catalogues of responsive analytes. This logic is relevant not only to high-performance sport but also to exercise medicine and rehabilitation, where the core task is to distinguish productive remodeling from delayed recovery and accumulating biological cost.

Progress in this field will not come from identifying more biomarkers, but from abandoning molecule-centric interpretation in favor of temporally structured, decision-linked frameworks that align signal kinetics with explicit monitoring decisions.

## Figures and Tables

**Table 1:** Candidate biomarker families across the recovery–overload continuum, emphasizing current translational maturity, interpretive value, and principal practical cautions within a decision-linked framework.

Family	Representative Signals	Typical Time Scale	Current Maturity and Main Interpretive Value	Best Application/ Main Caution
Fast load-sensitive signals	cfDNA; selected salivary stress markers; rapid metabolic changes	Minutes to a few hours	Moderate maturity for immediate biological cost; limited tissue specificity alone	Same-day load readout; not a delayed-recovery classifier
Delayed tissue-strain signals	CK; myoglobin; muscle-damage-linked metabolites; selected inflammatory proteins	6–48 h	Moderate maturity for tissue burden and incomplete recovery; large inter-individual variability	Eccentric or unusual load; interpret within athlete over 24–72 h
Regulatory signals	Circulating miRNAs; lncRNAs; EV cargo	Hours to days	Low-to-emerging maturity; closer to regulatory adaptation but analytically fragile	Mechanistic enrichment in repeated-measures research; not stand-alone
Integrated phenotype signals	Metabolomic fingerprints; combined multi-marker signatures	Context-dependent	Moderate promise when tightly timed and metadata-rich; overfitting risk in small cohorts	Panel reduction/ classification; external validation required

Notes: Time scales are approximate bout-relative windows and may shift with exercise mode, sampling matrix, training history, and recovery context. Translational maturity is expressed comparatively and refers to current readiness for repeated field monitoring rather than universal diagnostic validity. Representative signals are illustrative rather than exhaustive. cfDNA = cell-free DNA; CK = creatine kinase; EV = extracellular vesicle; lncRNA = long non-coding RNA; miRNA = microRNA. Comparative maturity, time-scale estimates, and principal cautions were synthesized from recent biomarker reviews, methodological standards, and the representative domain-specific literature [21,22,30,31,37,38,39,41,45,46,47,48,49,50,51,79,80,81].

**Table 2:** Purpose-specific examples of practical, decision-linked multi-marker panels, with suggested sampling windows and the specific operational interpretation each panel is intended to support.

Decision Goal	Minimal Panel	Suggested Sampling Window	Operational Interpretation
Acute training-load readout	Plasma cfDNA + salivary cortisol or alpha-amylase	Immediately post-bout and 1–3 h	Same-day biological cost and stress reactivity; not a delayed tissue-status readout
Delayed recovery/tissue-strain surveillance	CK or myoglobin + targeted metabolite panel + soreness/performance metric	24 h, with optional 48–72 h follow-up	Escalate concern when delayed biochemical strain coexists with symptoms or force loss
Adaptive signaling profile	Selected miRNAs/lncRNAs ± EV cargo + classical chemistry anchor	Same day plus 24–48 h repeated measures	Adds regulatory context to explain whether the pattern is resolving; analytically demanding
Research-grade precision profiling	Chemistry + metabolomics + cfDNA + RNA + metadata	Multi-time-point across bout and microcycle	Discovery and validation platform for smaller operational panels; vulnerable to cost and overfitting

Notes: The panels are illustrative examples intended for standardized repeated-measures monitoring anchored to within-athlete baselines. Suggested windows should be aligned with matrix-specific kinetics and the practical sampling context. The symbol ± indicates that EV cargo is optional rather than mandatory. Symptoms and performance should be interpreted alongside biochemical signals rather than replaced by them. Panel examples are illustrative and were assembled from translational workload-monitoring principles, decision-linked multi-marker logic, and representative biomarker-domain kinetics [21,22,32,45,46,47,48,72,86,88,90].

**Table 3:** Minimal reporting priorities for translational exercise biomarker studies.

Domain	Minimum Expectation	Why It Matters
Exercise stimulus	Report modality, intensity, duration, eccentric load, training status, and recovery interval	Without stimulus definition, biomarker meaning collapses
Sampling logic	State exact bout-relative collection times and time of day	Timing is part of the biology, not just logistics
Matrix handling	Describe collection device/tube, processing delay, centrifugation, storage, freeze–thaw history, and normalization	Pre-analytics can create or erase apparent biomarkers
Participant context	Report sex/hormonal status, age, diet or fasting, sleep, illness/medication use, and energy availability	Context explains heterogeneity and improves reproducibility
Analytical transparency	Specify assay platform, QC, missing-data handling, batch control, and multiple-testing control	High-dimensional results are uninterpretable without analytical discipline
Validation	Separate discovery from confirmation and benchmark against classical markers or performance outcomes	Panels must demonstrate added value, not novelty alone
Mechanistic anchor	State whether the signal is intended to represent acute load, tissue strain, regulatory adaptation, or inter-tissue communication	Prevents over-interpretation and improves panel design

Notes: These priorities are cumulative rather than alternative and apply to both classical and omics-based biomarker studies. Domain-specific standards may additionally require MIQE-, MISEV-, or MSI-consistent reporting and should be paired with exercise-specific reporting of stimulus, timing, and participant context. QC = quality control. Relevant paired reporting guidance includes consensus and methodological frameworks on sport load, recovery, and participant-context reporting on women [14,15,20,33].

## Data Availability

No new data were created or analyzed in this study. Data sharing is not applicable to this article.

## Abbreviations

cfDNA	Cell-free deoxyribonucleic acid
CK	Creatine kinase
CRP	C-reactive protein
EV	Extracellular vesicle
HIIT	High-intensity interval training
IgA	Immunoglobulin A
LDH	Lactate dehydrogenase
lncRNA	Long non-coding RNA
MC	Menstrual cycle
MISEV	Minimal Information for Studies of Extracellular Vesicles
MSI	Metabolomics Standards Initiative
RNA	Ribonucleic acid
AMPK	AMP-activated protein kinase
mTORC1	Mechanistic target of rapamycin complex 1
NF-κB	Nuclear factor kappa B
Nrf2	Nuclear factor erythroid 2-related factor 2
PGC-1α	Peroxisome proliferator-activated receptor gamma coactivator 1-alpha

## Appendix A. Literature Identification Strategy and Review Scope

This article was developed as a narrative review intended to integrate mechanistic, methodological, and translational evidence related to exercise-responsive biomarkers and their interpretation across the recovery–overload continuum. The objective was conceptual synthesis rather than exhaustive systematic aggregation of all exercise-responsive molecules. Accordingly, the decision-linked framework proposed in this review should be understood as an interpretive model derived from the integration of existing evidence rather than as a formally validated diagnostic system.

Literature identification was performed iteratively using PubMed, Scopus, and Web of Science and was complemented by the backward and forward citation tracking of key review articles, consensus statements, methodological papers, and foundational mechanistic studies. For transparency, the review scope prioritized the literature published from January 2000 to February 2026, while earlier landmark articles were retained selectively when they were necessary for historical context or mechanistic framing.

Searches combined three concept blocks using Boolean operators: (i) exercise exposure and recovery state; (ii) biomarker class or omics platform; and (iii) sampling matrix, kinetics, or monitoring context. Representative combinations included (exercise OR training OR athlete monitoring) AND (recovery OR overload OR overreaching OR overtraining) AND (biomarker* OR metabolomics OR “cell-free DNA” OR cfDNA OR “circulating RNA” OR microRNA OR lncRNA OR “extracellular vesicle*”) AND (blood OR plasma OR serum OR urine OR saliva OR “minimally invasive sampling”).

Additional focused searches were undertaken for pre-analytical issues, biomarker kinetics, sex and hormonal context, circadian timing, energy availability, workload-monitoring relevance, and validation or reporting standards. The review primarily considered the English-language literature from the broader exercise physiology, molecular exercise biology, and biomarker-methodology fields.

Primary human studies, consensus statements, methodological guidance documents, and translational monitoring studies were prioritized when they informed one or more of the following dimensions: bout-relative timing and kinetic behavior; sampling matrix considerations and pre-analytical constraints; linkage between biomarker signals and performance, recovery status, or symptoms; and feasibility for repeated monitoring in applied sport or exercise settings. Mechanistically informative non-human or tissue-level studies were used selectively when they helped clarify release biology, inter-tissue signaling, or interpretive kinetics relevant to translational inference.

Because the aim of the review is conceptual integration rather than quantitative synthesis, no formal risk-of-bias assessment, study-count flow diagram, or meta-analytic aggregation was performed. Judgments regarding translational maturity of biomarker domains therefore reflect interpretive synthesis of mechanistic evidence, methodological considerations, and practical monitoring relevance.
